# Treatment of a Subacute Locked Thumb Metacarpophalangeal Joint with Sesamoid Entrapment

**DOI:** 10.1155/2019/2136540

**Published:** 2019-03-24

**Authors:** John J. Carroll, William D. McClain, Julia A. V. Nuelle

**Affiliations:** Brooke Army Medical Center, Department of Orthopaedic Surgery, 3551 Roger Brooke Drive, Ft. Sam Houston, TX 78234, USA

## Abstract

**Introduction:**

Locked thumb metacarpophalangeal (MCP) joints due to entrapped radial sesamoids are rare injuries that commonly require open reduction, especially when the injury is delayed in presentation.

**Case Presentation:**

We present a case of a 24-year-old female with a subacute thumb MCP joint subluxation due to an incarcerated radial sesamoid. She underwent successful closed reduction but had persistent pain and difficulty gripping large objects necessitating eventual open volar plate repair despite therapy. She was able to achieve full motion, with little pain and disability, after undergoing delayed volar plate repair.

**Discussion:**

Delayed volar plate repair may be considered for those patients who fail to improve with conservative management and occupational therapy after a successful closed reduction for thumb MCP joint subluxation due to an incarcerated radial sesamoid.

## 1. Introduction

Subluxations of the thumb metacarpophalangeal (MCP) joint due to an entrapped radial sesamoid bone are rare injuries that often require open reduction, especially in the setting of a delayed presentation [1–4]. From our literature review, there are no cases describing patients who underwent successful closed reduction having a need for any operative procedures on the injured thumb in the future. We present a case of a subacute thumb MCP subluxation due to an incarcerated radial sesamoid that successfully underwent closed reduction at presentation. Due to persistent pain and decreased motion despite occupational therapy (OT), we obtained advanced imaging which demonstrated a volar plate tear. She eventually underwent an open volar plate repair in an attempt to improve these symptoms.

## 2. Case Presentation

**Figure 1:**
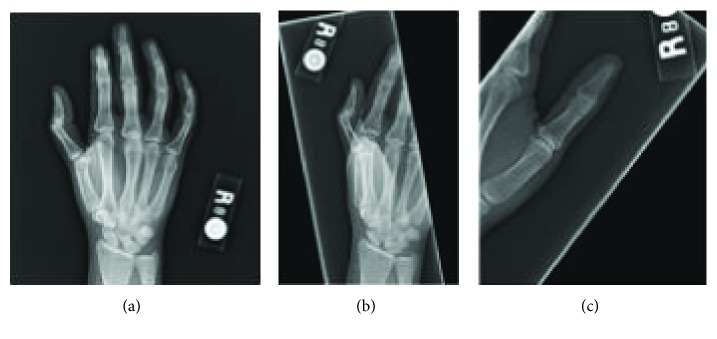
Injury radiographs. Obtained by urgent care on the day of injury. Radiology read stated no acute osseous abnormalities limited by patient positioning and pain due to decreased thumb range of motion. There is hyperextension present at the thumb MCP joint.

**Figure 2:**
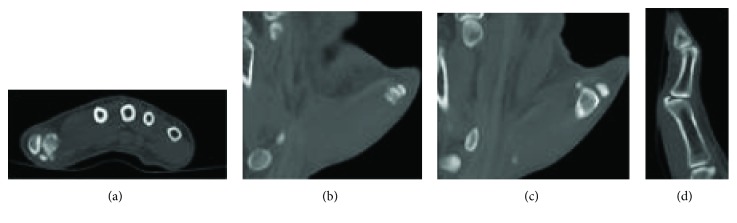
CT scan obtained three weeks after injury. Workup by primary physician included an ultrasound, which was nondiagnostic, and then this CT scan on the same day. Images demonstrated dorsal subluxation of the thumb MCP joint with incarcerated radial sesamoid.

A 24-year-old right hand dominant active duty marine canine trainer sustained a hyperextension injury to her right thumb with immediate onset pain and decreased range of motion. The patient presented to an outside urgent care the day after the injury where radiographs were obtained that were limited secondary to her positioning and pain. The provider noted no dislocation or subluxation at the time (Figure 1). At three weeks following her injury, a computed tomography (CT) scan obtained by her primary provider demonstrated dorsal subluxation of her right thumb MCP joint with entrapment of the radial sesamoid (Figure 2). She was then referred to our Emergency Department where she was evaluated by our team.

**Figure 3:**
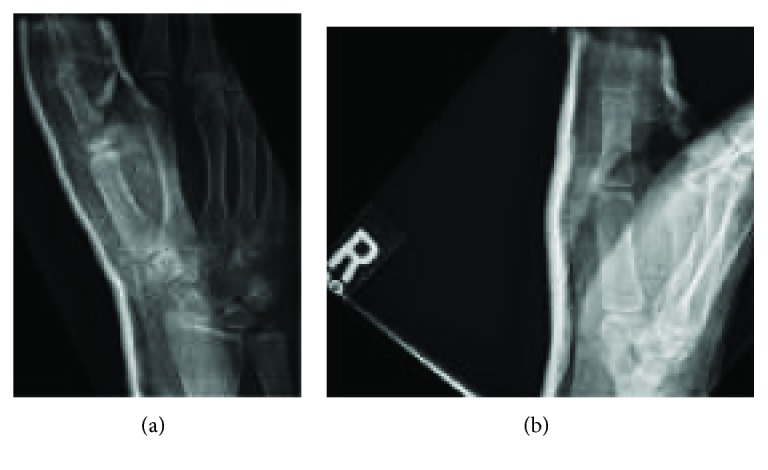
Closed reduction. The patient was referred to the Emergency Department where she was evaluated by orthopaedics and underwent insufflation of the MCP joint and closed reduction. The patient was noted to be unstable with greater than 10° of extension. The patient maintained in this splint for one week until first follow-up appointment.

On exam, the patient maintained her thumb MCP joint in approximately 30° of hyperextension. She was unable to flex beyond this due to pain and a mechanical block to motion. Using sterile technique and the assistance of fluoroscopy, 1% Lidocaine without epinephrine was injected into the thumb MCP joint, entering from the dorsal and radial side. Closed reduction was successfully performed with initial hyperextension and then forced flexion with this force directed at the dorsal base of the proximal phalanx. The patient's joint was noted to have volar instability at approximately 10° of extension. Her thumb was splinted in a reduced position at neutral flexion/extension (Figure 3).

The patient was evaluated in a clinic one week later, and radiographs in the splint demonstrated maintained reduction. The splint was removed, and the patient's joint was noted to be stable through full passive range of motion. She was transitioned to a custom OT hand-based thumb spica splint and started on gentle motion as well as grip/pinch strengthening exercises with OT. The patient transitioned out of the splint approximately six weeks after the reduction and was allowed to weight bear as tolerated.

At seven weeks after reduction, initial Quick Disabilities of the Arm, Shoulder, and Hand (QuickDASH) and grip strength measurements were recorded. The QuickDASH score was 45.45. Strength was decreased in the right thumb compared to the left with neutral grip (right: 60 pounds (lbs), left: 75 lbs), lateral pinch (right: 11 lbs, left: 17 lbs), 2-pt pinch (right: 7 lbs, left: 10 lbs), and 3-jaw pinch (right: 12 lbs, left: 18 lbs). Thumb MCP motion was 0-35°, symmetric to the left thumb. The patient reported pain with motion and gripping objects and plans were made for continued OT.

**Figure 4:**
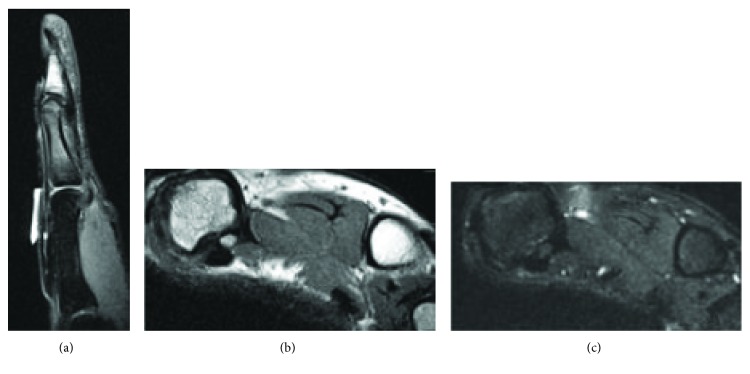
MRI obtained three months after reduction. MRI obtained 3 months after reduction demonstrated rupture of the thumb MCP volar plate with intact radial and ulnar collateral ligaments.

At 11 weeks after reduction, the patient reported some improvement in pain with motion but continued to have pain with grasping larger objects and had worsening motion. She had difficulty performing her tasks as an active duty marine and had persistent pain limiting her quality of life. QuickDASH score was 27.27. Right thumb grip strengths were now neutral grip 70 lbs, lateral pinch 14 lbs, 2-pt pinch 10 lbs, and 3-jaw pinch 15 lbs, which were nearly symmetric to contralateral thumb. Active thumb motion was 0-24°. The patient was encouraged to continue with OT, and a magnetic resonance imaging (MRI) was ordered. The MRI demonstrated a reduced MCP joint without soft tissue interposition, but it also demonstrated of a volar plate rupture (Figure 4). Treatment options were reviewed with the patient to include volar plate repair, and she wished to proceed.

**Figure 5:**
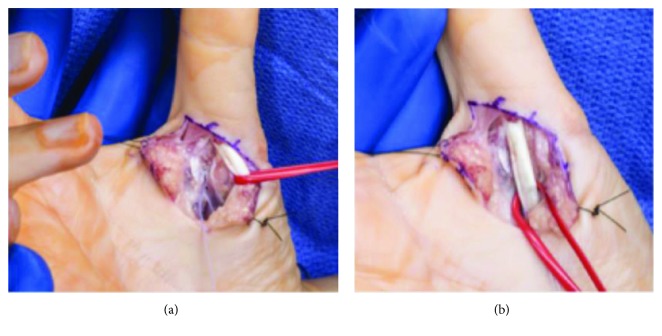
Clinical photographs of the right thumb volar plate repair. (a) Image demonstrates retracted flexor tendon with suture tails exiting from suture anchor inserted into the thumb metacarpal. (b) Image demonstrates flexor tendons resting over the repaired volar plate.

Approximately three and a half months after successful closed reduction, the patient underwent right thumb MCP joint volar plate repair. The sesamoids remained attached to the volar plate, and the volar plate was avulsed proximally from the metacarpal. The volar plate was not entrapped within the joint. A Mitek micro anchor was used to repair the volar plate and secure it to the subcondylar fossa of the metacarpal (Figure 5). She was noted to have some chondromalacia of the MCP joint. The cartilage surfaces of the sesamoids were intact, and both sesamoids were left in place. The patient's thumb was stable through full range of motion without any gapping under fluoroscopy with stress examination. She remained in the thumb spica splint for four weeks postoperatively prior to beginning gentle motion with OT. At two months postoperatively, the patient had active thumb MCP joint motion from 0-30°. At three months postoperatively, she had symmetric full range of motion and only had complaint of mild pain. At four months postoperatively, the patient reported continued improvement in pain and had a QuickDASH score of 9.01. She also subjectively reported that the pain she had when gripping larger objects had improved. She reached her separation time from the military and was lost to clinical follow-up as she moved from the local area. We were able to make contact with the patient electronically at one year postoperatively, and she was doing very well with a QuickDASH score of 4.55.

## 3. Discussion

The differential for locked thumb MP joint includes entrapment of volar plate, sesamoid, fracture fragments, locked trigger thumb, osteophytes, and cartilage defects on metacarpal head [5]. Incarceration of the radial sesamoid is relatively rare, and most reports of this entity have come out of the Japanese literature. Tsuge and Watari published one of the earliest case series on this subject in 1974 and described seven cases with six of them requiring open reduction [4]. Around a similar time period, Kojima and his colleagues compiled 29 cases of locked thumb MCP joints and detailed their successful nonoperative treatment. They concluded that the incarceration of a sesamoid in the joint space was anatomically impossible due to the fact that the sesamoids are imbedded in the volar plate of the MCP joint of the thumb [6]. Since then, there have been several authors who have published cases detailing incarcerated sesamoids in the thumb MCP joints requiring open reduction at varying times from injury, including Cheng et al. on the day of injury, Zhang et al. at five days, Desai and Morgan at two weeks, and Izadpanah and Wanzel at two months from injury [1–3, 7].

It is accepted that a hyperextension force contributes to the injury, but authors have investigated the pathoanatomy of this rare injury. Desai and Morgan noted that when the attachment between the sesamoid and volar plate is intact, it was impossible to bring the radial sesamoid into the joint space because of lack of excursion of the volar plate. In order to reproduce this injury, the volar plate had to be avulsed proximally to orient the radial sesamoid in a perpendicular manner so that entrapment can occur. Desai and Morgan additionally noted difficulty in reproducing the locking in cadaveric specimens and hypothesized the importance of a dynamic component of this injury, specifically contraction of the flexor pollicis brevis with tension on the intact radial collateral ligament [1]. Similarly, Xiong et al. described a mechanism by which the dynamic contributions from the flexor pollicis brevis and abductor pollicis during attempted flexion across the MCP joint cause slight hyperextension. This dynamic contribution is thought to be a secondary stress to the trauma, which resulted in the radial sesamoid becoming incarcerated within the joint [8].

There is no agreement with regard to the best treatment for the injury. Closed reduction should be attempted but is often unsuccessful in those patients with delayed presentation based on our limited literature. The classic reduction maneuver is described as hyperextension of the MCP joint with direct pressure on dorsal base of proximal phalanx. The key is to avoid axial traction alone as this may allow the volar plate to enter into the joint space, blocking reduction. Closed reduction may be aided by insufflation of the MCP joint with fluid to distend joint capsule [9]. In those patients who have failed closed reduction, open reduction should be performed. In literature, varying procedures have been performed in addition to open reduction of the MCP joint. Zhang et al. described radial sesamoid excision and volar plate repair, and Xiong et al. described cutting the insertion of the abductor pollicis brevis and flexor pollicis brevis at the base of the proximal phalanx [3, 8]. Desai and Morgan recommended excision of the radial sesamoid only in the presence of a groove-like depression in the metacarpal head or significant stripping of the sesamoid from the volar plate [1]. We surmise that volar plate repair benefited our patient even though the sesamoid was no longer entrapped after reduction and the volar plate remained out of the joint, since the volar plate did not properly heal with nonoperative management. This likely led to abnormal forces across the metacarpophalangeal joint and certain maneuvers resulted in stretching of the torn volar plate. These likely contributed to the patient's pain and difficulty grasping larger objects.

Our case is unique in the sense that the patient underwent successful closed reduction after having a subacute injury. She maintained this reduction and had some improvements with therapy but had persistent pain and decreased motion. There have been no described cases of patients needing operative procedures after successfully undergoing closed reduction. Our patient underwent the open volar plate repair three and a half months after successful closed reduction and nearly four and a half months after injury, which resulted in a return to baseline motion with minimal pain.
